# Band Dynamics of Multimode Resonant Nanophotonic Lattices with Adjustable Liquid Interfaces

**DOI:** 10.3390/nano13162350

**Published:** 2023-08-16

**Authors:** Nasrin Razmjooei, Robert Magnusson

**Affiliations:** Department of Electrical Engineering, University of Texas at Arlington, Arlington, TX 76019, USA; nasrin.razmjooei@mavs.uta.edu

**Keywords:** bound-state transitions, guided-mode resonance, band dynamics, subwavelength resonant lattice, nanophotonics, metamaterials

## Abstract

Subwavelength resonant lattices offer a wide range of fascinating spectral phenomena under broadside illumination. The resonance mechanism relies on the generation of lateral Bloch modes that are phase matched to evanescent diffraction orders. The spectral properties and the total number of resonance states are governed by the structure of leaky modes and the mode count. This study investigates the effect of interface modifications on the band dynamics and bound-state transitions in guided-mode resonant lattices. We provide photonic lattices comprising rectangular Si_3_N_4_ rods with a liquid film with an adjustable boundary. The band structures and band flips are examined through numerical simulations using the rigorous coupled-wave analysis (RCWA) method and analyzing the zero-order spectral reflectance as a function of the incident angle. The band structures and band flips are examined through numerical simulations, and the influences of the refractive index and the thickness of the oil layer on the band dynamics are investigated. The results reveal distinct resonance linewidths corresponding to different refractive indices of the oil layer. Furthermore, the effect of the oil thickness on the band dynamics is explored, demonstrating precise control over the number of propagating modes within the lattice structure. Theoretical simulations and experimental results are presented for a subwavelength silicon-nitride lattice combined with a liquid film featuring an adjustable boundary. The presence of a relatively thick liquid waveguiding region enables the emergence of additional modes, including the first four transverse-electric (TE) leaky modes, which produce observable resonance signatures. Through experimental manipulation of the basic lattice’s duty cycle, the four bands undergo quantifiable band transitions and closures. The experimental results obtained within the 1400–1600 nm spectral range exhibit reasonable agreement with the numerical analysis. These findings underscore the significant role played by the interface in shaping the band dynamics of the lattice structure, providing valuable insights into the design and optimization of photonic lattices with adjustable interfaces.

## 1. Introduction

The interaction of electromagnetic waves with periodic media has long been a subject of interest in photonics. When the period and wavelength are on similar scales, the attendant band structure plays a crucial role in governing the propagation of electromagnetic waves, including light. The band structure defines a range of frequencies, known as the band gap, in which certain frequencies of electromagnetic waves cannot propagate through a periodic material or structure along a specific direction [1].

In photonics, periodic variation in the dielectric constant gives rise to the band structure, resulting in energy bands and band gaps. The ability to engineer these bands in periodic photonic structures offers opportunities for controlling light at nano- or microscales. One intriguing phenomenon that arises from lattice–light interactions is leaky-mode or guided-mode resonance. This occurs when an incident light wave phase matches a lateral Bloch mode supported by the optical lattice. Unlike three-dimensional photonic crystal Bragg-type stop bands, these resonant lattices operate in the second stop band and possess an out-of-plane radiative energy-coupling channel. As a result, the leaky mode resides above the light line in the Brillouin zone.

The physical and spectral properties of resonant lattices are strongly influenced by their symmetry. For symmetrical lattices, only one edge of the second stop band exhibits resonance and attendant radiation, while both edges resonate and radiate for asymmetric lattices. The nonradiant edge, known as the “bound state in the continuum” (BIC), is protected by symmetry and cannot radiate, even if its frequency is above the light line and connected with the radiation continuum [2,3,4]. Optical BICs were introduced in 2008 to describe this blocked resonance at the nonleaky edge [5], drawing on analogies with bound states in quantum systems [6]. Since then, the term BIC has gained wide acceptance and has shed light on the guided-mode resonant (GMR) effects in the rapidly growing fields of metamaterials and metasurfaces. The physics and potential applications of resonant lattices and their leaky-edge BIC states have become subjects of significant scientific interest [7,8,9,10,11,12,13,14,15,16,17,18,19,20,21]. Tailoring light–matter interactions is not limited to the dielectric system. In recent years, the field of enhancing light–matter interactions has witnessed remarkable progress through the exploration of novel strategies involving photonic crystals coupled to plasmonic nanoparticles. These hybrid systems offer a unique platform for tailoring and amplifying optical interactions, leading to solid prospects in various scientific and technological domains. For instance, Barreda et al. [22] presented a study on hybrid photonic–plasmonic cavities, leveraging the nanoparticle-on-a-mirror configuration to achieve enhanced light confinement and the excitation of resonant modes. Moreover, the work by Palstra et al. [23] introduced hybrid cavity–antenna systems designed to facilitate quantum optics studies beyond the confines of traditional cryostats.

Previous research has indicated the possibility of interchanging the frequency locations of leaky and nonleaky edges in periodic films [4]. Perturbations such as changes in the refractive index or geometry of a resonant structure can dramatically alter the frequency position and shape of its photonic bands. Studies have shown that periodic lattice dielectric functions can exhibit interchanged frequency locations of leaky and nonleaky edges by varying the duty cycle or dielectric lattice contrast [21]. Analytical coupled-wave theory models have established that the bands close at certain points where the coefficients of the dielectric function’s Fourier harmonics are related in particular ways [4,24,25]. By making appropriate design choices, it becomes possible to achieve closed-band states and band flips, where the leaky and nonleaky edges exchange positions. This intriguing behavior of band dynamics and bound-state transitions forms the basis of the present study.

Prior investigations have explored the band structure and band flip properties of resonant lattices in simple photonic structures, including one-dimensional dielectric photonic lattices [21]. These studies have demonstrated that the behavior of band flips can be controlled by tuning the geometrical parameters of the structure, such as the lattice contrast and fill factor. In our previous work [26], we experimentally demonstrated band flips and bound-state transitions in one-dimensional dielectric photonic lattices composed of a photoresist grating, a Si_3_N_4_ sublayer, and a glass substrate with distinct fill factors. We provided a comprehensive understanding of the physical processes underlying the positioning of leaky/blocked (GMR/BIC) edges and the band closure point, shedding light on the relevant physics. The designed photonic lattices were fabricated and characterized, and a comparison between the theoretical results and experimental data demonstrated a reasonable agreement. This study offered valuable insights into the band dynamics of simple dielectric photonic lattices.

In this paper, we expand our understanding of resonant lattice systems by delving into the more complex behavior of multiple simultaneous bands by introducing a liquid–medium interface. We investigate the effects of interface modifications, such as changes in the refractive index or thickness, on the band dynamics of resonant lattices. The adjustable interface within the lattice structure enables precise control over the number of propagating modes and offers new possibilities for manipulating light at the nanoscale. The novelty of the current study lies in the design and experimental quantification of new resonant lattice systems that are capable of supporting multiple leaky modes. By introducing an adjustable interface within the multimode waveguide structure, we can manipulate the number of modes propagating within the structure. This adjustability allows us to control the mode propagation properties, resulting in the emergence or suppression of specific resonant modes.

The results from this study contribute to the advancement of photonic devices with adjustable interfaces, with potential applications in optical filters, sensors, and other resonance-based technologies. 

## 2. Theory and Numerical Results

**Figure 1:**
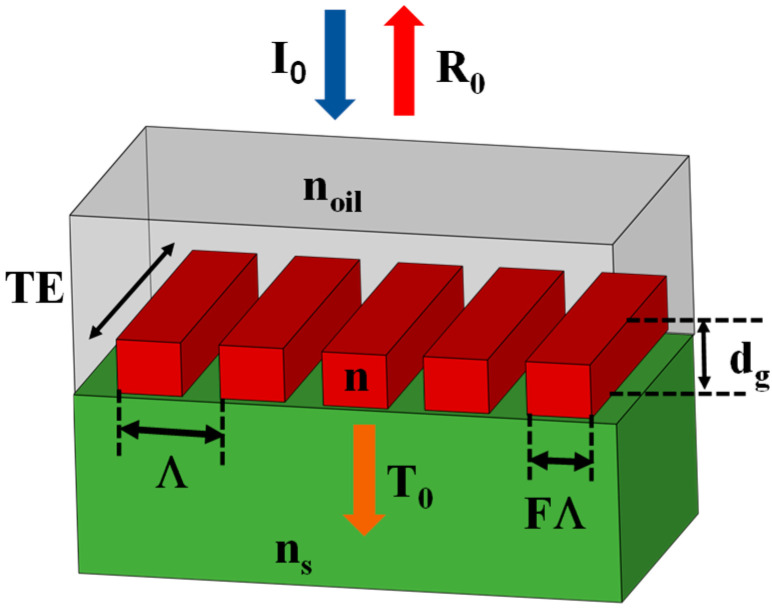
Model design comprising rectangular Si_3_N_4_ rods with a refractive index (*n*) of 1.8, a thickness (*d_g_*) of 2000 nm, and a period (Λ) of 1000 nm. The lattice is immersed in index-matching oil with a refractive index of 1.53 and positioned on a glass substrate with a refractive index of 1.5. The fill factor F represents the fraction of the period that is occupied by the grating material.

Figure 1 shows a conceptual device made from rectangular Si_3_N_4_ rods with a refractive index (*n*) of 1.8, a thickness (*d_g_*) of 2000 nm, and a period (Λ) of 1000 nm. The lattice is immersed in index-matching oil with refractive index of 1.53 and placed on a glass substrate with a refractive index of 1.5. 

Corresponding band structures and band flips for the design in Figure 1 are illustrated using numerical methods based on a rigorous coupled-wave analysis (RCWA) by calculating the zero-order spectral reflectance as a function of the incident angle. RCWA is a fundamental computational technique that is extensively employed in photonics and diffraction studies to rigorously analyze the interaction of electromagnetic waves with periodic structures. RCWA systematically solves Maxwell’s equations within periodic media by decomposing the electromagnetic field into its constituent Fourier components and coupling these components. This approach treats periodic structures using superpositions of plane waves with varying wave vectors. Determining the attendant space–harmonic amplitudes permits the calculation of the diffraction efficiencies of all diffraction orders. As a result, RCWA facilitates the accurate prediction of external optical power flow, resonant modes, and band structures in complex photonic systems, such as gratings, photonic crystals, and metamaterials. Its capacity to handle intricate structures and provide insights into optical properties positions RCWA as a vital tool in modern photonics research and engineering. Excellent references with full detailed explanations and formulations include [27,28]. The calculated results presented herein are found by use of the Synopsys DiffractMOD numerical tool set [29].

**Figure 2:**
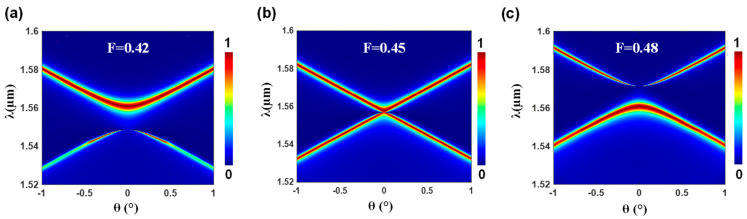
The zero-order spectral reflectance as a function of the incident angle for the design shown in Figure 1 with distinct fill factors (*F*) of (**a**) *F* = 0.42, (**b**) *F* = 0.45, and (**c**) *F* = 0.48. The incoming wave is polarized in the TE state, and its electric field vector is aligned with the grating ridges. It should be noted that the upper and lower edges refer to shorter and longer wavelengths, respectively, as is conventionally understood in frequency terms. The substrate and cover are modeled as infinite regions.

The band flip is achieved for fill factors (*F*) of 0.42, 0.45, and 0.48, as shown in Figure 2. When the *F* is 0.42, the band gap is open with a nonleaky (BIC) edge and a leaky (GMR) edge at the upper and lower frequency bands, respectively. After raising *F* to 0.45, the band closes and then reopens at 0.48 with the BIC-GMR edges interchanged. 

The addition of the index-matching liquid to the silicon nitride grating creates a structure with a waveguide layer. This waveguide layer enables the occurrence of guided-mode resonance in the system. By introducing the index-matching liquid, we create a quasi-membrane-like structure, where the combination of the grating and the liquid layer supports guided-mode resonances. The purpose of incorporating the index-matching liquid in our study is to create a waveguide layer that allows for the excitation and confinement of guided-mode resonances within the structure in the chosen wavelength region. If we remove the matching layer, the effective mode index drops, and the region of resonance shifts to a shorter band.

## 3. Impact of Interface Modifications on the Band Dynamics of Photonic Lattices

**Figure 3:**
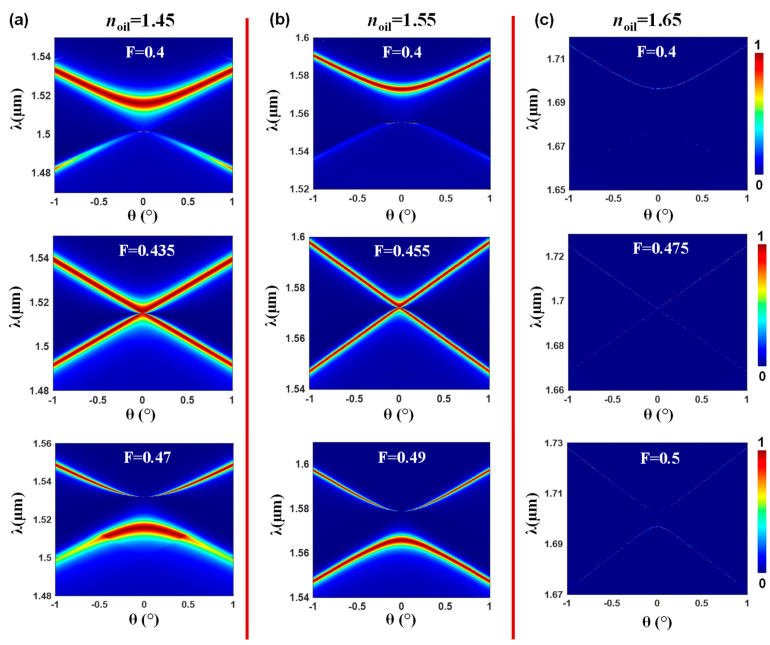
Effect of the liquid interface refractive index on the band dynamics of the photonic lattice. (**a**) Band dynamics of the lattice with *n_oil_* = 1.45, demonstrating three different fill factors: *F* = 0.4, *F* = 0.435, and *F* = 0.47. (**b**) Band dynamics of the lattice with *n_oil_* = 1.55, *F* = 0.4, *F* = 0.455, and *F* = 0.49. (**c**) Band dynamics of the lattice with *n_oil_* = 1.65, *F* = 0.4, *F* = 0.475, and *F* = 0.5. The substrate and cover are modeled as infinite regions.

In the subsequent analysis, we extend our investigation to explore the influence of the refractive index of the index-matching oil layer on the band dynamics of the lattice depicted in Figure 1. Specifically, we demonstrate the band dynamics results for three different refractive indices of the oil layer: *n_oil_* = 1.45, *n_oil_* = 1.55, and *n_oil_* = 1.65. Our results, which are presented in Figure 3, reveal distinct characteristics, including the resonance linewidths and spectral locations corresponding to different refractive indices of the oil layer. Notably, the band structure associated with *n_oil_* = 1.45 exhibits the broadest resonance linewidth among all examined oil indices. On the other hand, the band structure for *n_oil_* = 1.65 displays a considerably narrower linewidth. These results align with our earlier works, which established that larger refractive index differences between the grating and the surrounding medium lead to broader resonance linewidths.

**Figure 4:**
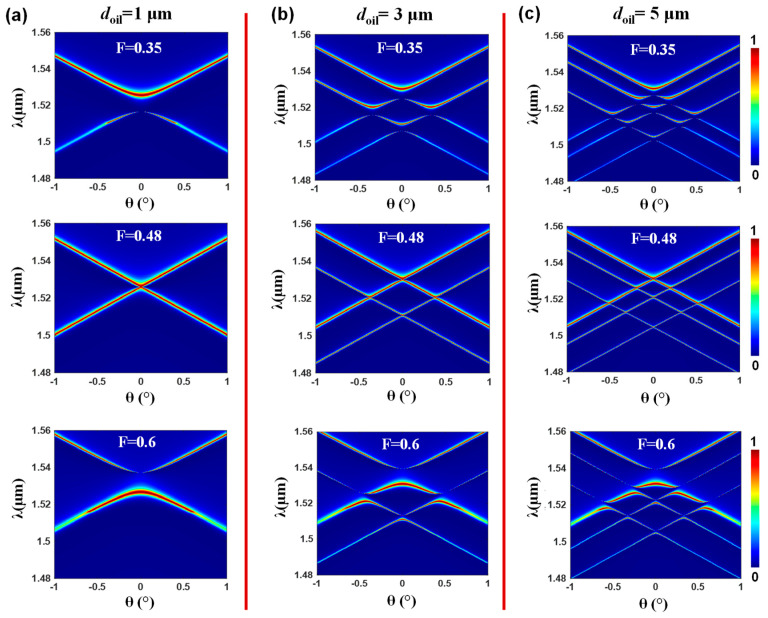
Effect of the liquid interface thickness on the band dynamics of the photonic lattice (**a**) Band dynamics of the lattice with a thickness of oil (*d_oil_*) set to 1 μm, demonstrating the propagation of the fundamental mode. (**b**) Band dynamics of the lattice with a *d_oil_* value of 3 μm, showcasing the emergence and propagation of two modes, TE_0_ and TE_1_. (**c**) Band dynamics of the lattice with a *d_oil_* value of 5 μm, illustrating the accommodation of three modes: TE_0_, TE_1_, and TE_2_. The simulation employs three different fill factors, namely *F* = 0.35, *F* = 0.48, and *F* = 0.6. The substrate is modeled as an infinite region, whereas there is an air/liquid interface on the top side.

After investigating the influence of the oil index on the band dynamics, we now shift our focus to examining the effect of the oil thickness on the band dynamics of the device. The designed structure features parameters of *d_g_* = 0.2 μm, Λ = 1 μm, *n_s_* = 1.5, and *n_oil_* = 1.53, with the thickness of the oil layer varied as *d_oil_* = 1 μm, *d_oil_* = 3 μm, and *d_oil_* = 5 μm. The simulation utilizes three different fill factors, including *F* = 0.35, *F* = 0.48, and *F* = 0.6. Our results presented in Figure 4 demonstrate that when the oil-film thickness is at its lowest value of 1 μm, only the fundamental mode TE_0_ is capable of propagating within the structure (Figure 4a). As we increase the thickness to 3 μm (Figure 4b), two additional modes, namely TE_0_ and TE_1_, emerge and propagate within the design. Finally, when the oil thickness reaches 5 μm, the structure accommodates three modes, TE_0_, TE_1_, and TE_2_, as shown in Figure 4. This indicates that, by tuning the thickness of the oil interface layer, precise control over the number of propagating modes within the lattice can be achieved. If the refractive index of the liquid is decreased, thereby reducing the air/liquid refractive index contrast, there will be weaker modal guidance and thus fewer modes allowed. These findings underscore the significant role played by the oil thickness in shaping the band dynamics of the lattice structure. Moreover, the attendant spectral variety and control are shown.

## 4. Experimental Verification

The experimental setup for studying the band dynamics involves the utilization of Si_3_N_4_ (*n* = 1.8) with a thickness of *d_g_* = 250 nm deposited on a glass substrate (*n_s_* = 1.5). Index-matching oil (*n_oil_* = 1.53) with a thickness of *d_oil_* = 10 μm is employed to achieve the desired experimental conditions. The fabrication process begins by cleaning the quartz substrate wafer and depositing a thin layer of Si_3_N_4_ using plasma-enhanced chemical vapor deposition (PECVD). Subsequently, 1D Si_3_N_4_ grating patterns are created through UV laser interference lithography and a dry etching process. A photoresist (PR, Shipley 1813) layer is spin-coated and exposed to a coherent beam (λ = 266 nm) using a Lloyd’s mirror setup to achieve the desired fill factor. The Si_3_N_4_ layer is then etched using a reactive-ion etcher (Oxford Plasmalab 80 Plus) with a CHF_3_ + SF_6_ gas mixture. To optimize the etch rate, selectivity, and anisotropy of the device profile, a specific Si_3_N_4_ etch recipe was developed for this study. After the reactive-ion etching, a thin layer of residual PR remains on the grating, which is eliminated by performing O_2_ plasma ashing for 8 min. Following the removal of residual PR, a Si_3_N_4_ 1D grating is formed on the glass substrate.

**Figure 5:**
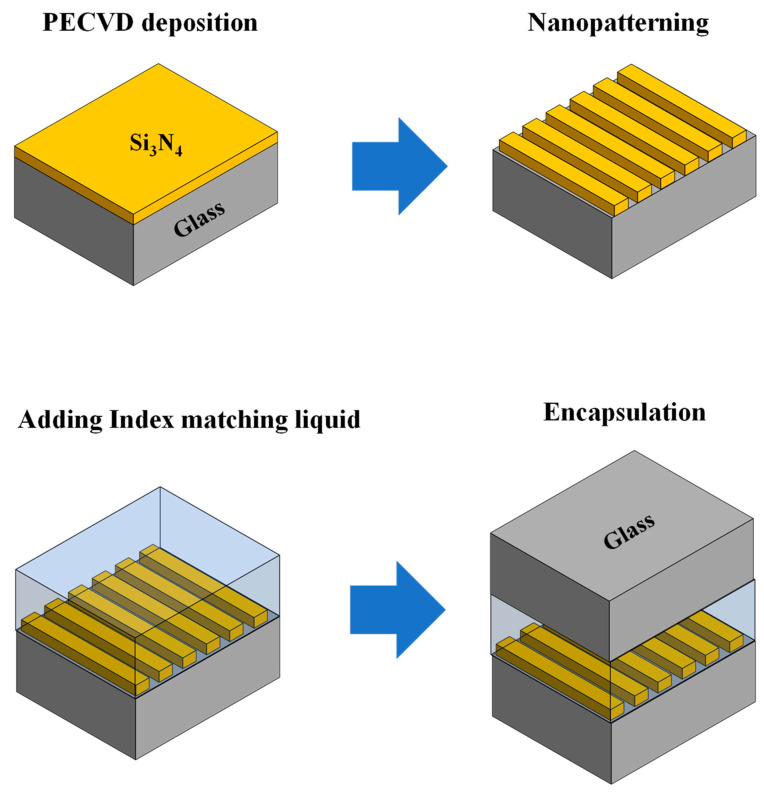
Schematic diagram illustrating the fabrication process of the multimode guided-mode resonant structure.

Subsequently, the Si_3_N_4_ grating is immersed in refractive-index-matching oil (Cargille Lab. Series A), which is a liquid employed to match the refractive index of two materials with different properties. The design is then encapsulated using a glass cover. Due to the higher refractive index of the oil compared to the glass, an effective waveguiding film is created, enabling the presence of additional quasi-guided leaky modes in the structure. To illustrate the fabrication process of the Si_3_N_4_/oil guided-mode resonant structure, a schematic diagram is provided in Figure 5.

In a previous study [26], we described the experimental configuration employed for measuring the optical transmission of the resonant grating. This setup involves the propagation of light emitted by a supercontinuum laser source through an optical fiber and a collimator. Subsequently, the light passes through a polarizer and an optical aperture before reaching the lattice structure. The transmitted light is quantified using a detector and an optical spectrum analyzer (OSA), with data collection spanning wavelengths from 1480 nm to 1580 nm and incident angles ranging from −1° to 1° at increments of Δθ = 0.01°. The reflection spectrum is obtained by subtracting the zero-order transmittance (T_0_) from unity (R_0_ = 1 − T_0_), and the acquired data are processed using MATLAB. This method is applied here as well. The measurements are conducted for all three fabricated devices.

**Figure 6:**
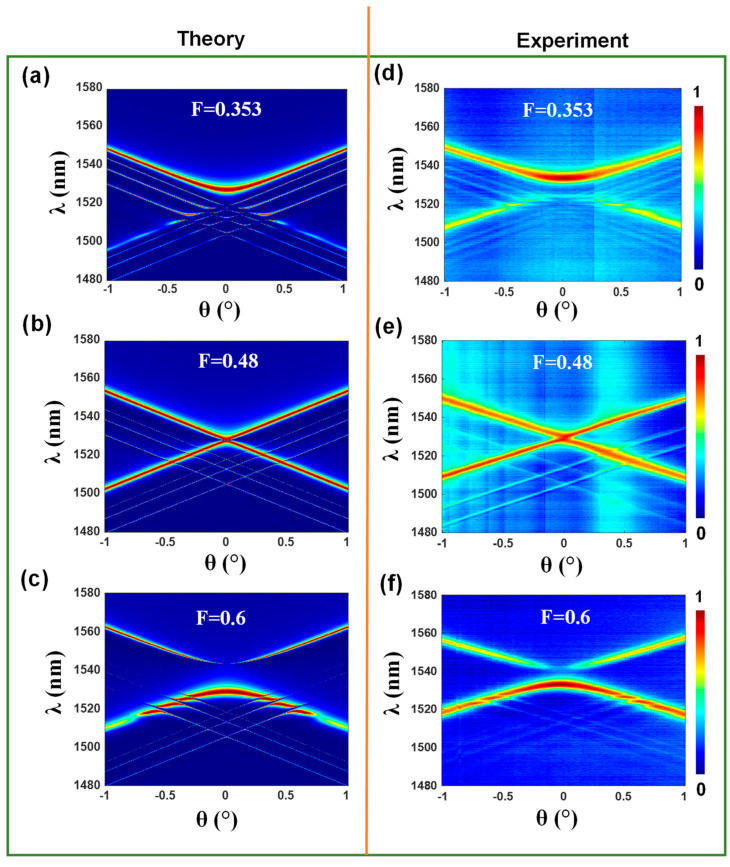
Theoretical and experimental results for devices with varying *F* values. (**a**–**c**) shows the simulation results for *F* values of 0.353, 0.48, and 0.6, respectively, while (**d**–**f**) shows the corresponding experimental data. The figure demonstrates the multimode band structure and band flip effects observed in both the simulation and experimentation, as well as the transition from the nonleaky BIC state to the leaky GMR state across the bandgap.

Figure 6 presents both the theoretical simulations and the corresponding experimental results obtained for the fabricated devices. The simulation results for the fill factors of *F* = 0.353, *F* = 0.48, and *F* = 0.6 are depicted in Figure 6a–c, respectively. Similarly, Figure 6d–f presents the experimental data for the same device structures. The figure illustrates that the fabricated devices demonstrate band structure and band flip effects that exhibit broad agreement with the simulated results. Furthermore, the experimental data illustrate the transition from the nonleaky bound-in-continuum (BIC) state to the leaky guided-mode resonant (GMR) state within the bandgap.

## 5. Discussion

The zero-order reflection map, as a function of the wavelength and angle, is obtained for the symmetric Si_3_N_4_ grating immersed in index-matching oil. The maps reveal multiple band structures resulting from the additional interface introduced by the oil layer and the attendant formation of a waveguide region. Notably, Figure 6 showcases the emergence of multiple experimental resonant leaky mode bands with the primary resonance lines corresponding to the fundamental mode TE_0_. The TE_0_ mode exhibits the highest efficiencies, as it interacts most strongly with the high-index film, resulting in a larger linewidth. There is approximate agreement in the wavelength locations of the experimental and theoretical spectra for this mode. The lines for the higher modes are narrower and the spectra are weaker due to being limited by the experimental setup and its resolution of Δλ = 0.05 nm. The incident Gaussian beam has a 1 mm diameter and attendant divergence, additionally limiting the narrow-line observability somewhat. 

## 6. Conclusions

In conclusion, this comprehensive study examined the band dynamics of distinct photonic lattice designs. We investigated the effects of the refractive index and thickness of the liquid–film interface on the band dynamics of the presented design, revealing their significant influences on the resonance linewidths, spectral placement, and the number of propagating modes within the lattice structure. Additionally, we analyzed the band structure and band flip phenomena in a subwavelength silicon-nitride lattice integrated with a liquid film with a tunable boundary. This investigation highlighted the emergence of multiple resonant leaky mode bands and the formation of a waveguide region due to the presence of the liquid layer. Notably, we observed a band flip not only in the band structure associated with the fundamental mode but also in the higher-order modes. Furthermore, by modifying the fill factor of the grating, we demonstrated the ability of the proposed lattice structure to induce transitions for each propagating mode. The lattice design supported the propagation of the fundamental TE_0_ mode, as well as higher-order modes such as TE_1_, TE_2_, and TE_3_. The results emphasize that the resonance lines with the highest efficiencies were primarily attributed to the fundamental mode TE_0_, which exhibited the strongest interaction with the high-index film. The experimental demonstration showcasing the band flip phenomenon under multimode propagation exhibited satisfactory agreement with the provided numerical results.

Moving forward, further research can delve into more complex lattice designs and explore additional parameters and their interactions to enhance our understanding of the underlying physics and expand the possibilities for tailored band engineering. The knowledge gained from such investigations will contribute to the development of advanced photonic devices with improved performance and functionality.

## Data Availability

The data that support the findings of this paper are available from the corresponding author upon reasonable request.
